# Chitosan Based Heterogeneous Catalyses: Chitosan-Grafted-Poly(4-Vinylpyridne) as an Efficient Catalyst for Michael Additions and Alkylpyridazinyl Carbonitrile Oxidation

**DOI:** 10.3390/molecules18055288

**Published:** 2013-05-08

**Authors:** Khaled D. Khalil, Hamad M. Al-Matar

**Affiliations:** 1 Chemistry Department, Faculty of Science, University of Kuwait, P.O. Box 5969, Safat 13060, Kuwait; 2 Chemistry Department, Faculty of Science, Cairo University, Giza 12613, Egypt

**Keywords:** chitosan, chitosan-*g*-poly(4-vinylpyridine), graft copolymer, [chitosan-*g*-PVP]-supported iron (III) complex, Michael additions

## Abstract

Chitosan-grafted-poly(4-vinylpyridine) (Cs-PVP) copolymers could be synthesized under heterogeneous conditions in presence of a potassium persulfate and sodium sulfite redox system. The synthesized graft copolymer could be utilized effectively, in the form of beads, as an efficient catalyst for Michael additions of active methylenes to functionally substituted alkenes. Moreover, methyl moiety oxidation in methyl pyridazinyl carbonitriles by H_2_O_2_ in the presence of chitosan-g-polyvinyl pyridine–supported iron (III) complex, Cs-PVP/Fe, could be affected. A variety of pyrans, naphthopyrans, and thiopyrans could be synthesized efficiently in the presence of these graft copolymer beads by novel catalytic routes. These polymeric catalysts could be used instead of the old toxic commercial organic basic catalysts, piperidine or pyridine, and could be readily isolated from the reaction mixture and recycled several times without significant loss of catalytic activity.

## 1. Introduction

**Scheme 1:**
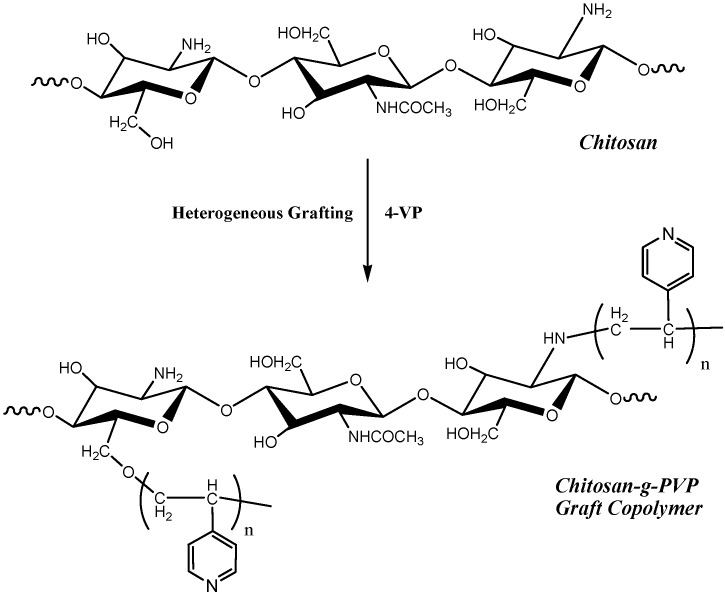
Preparation of chitosan-g-PVP catalyst.

Recently, biocatalysis and sustainable chemistry researchers are directed to develop new green methodologies that aim to reduce and prevent pollution at its source [1,2,3]. Biocatalysis is a promising technique based on the use of natural renewable biological materials, such as enzymes and polymers, that provide cleaner methodologies with high selectivity and energy-efficient operation under mild conditions in contrast to the traditional chemical catalysts [4,5]. Chitosan (2-acetamido-2-deoxy-β-D-glucose-(N-acetylglucosamine) is a partially deacetylated polymer of chitin and is usually prepared from chitin by reflux with a strong alkaline solution [6,7,8]. Some time ago, the utility of chitosan as an efficient eco-friendly basic biocatalyst for Michael additions was reported and it could already be shown that chitosan can catalyze the addition of bi-functional active methylenes to arylidenemalononitriles and enaminones [9,10,11,12]. This type of reaction, involving simple addition of active methylenes, phenols and naphthols to electron poor functionally substituted alkenes, is an efficient C-C bond forming reaction. Classically these additions have been effected in the presence of homogeneous basic catalysis [13,14,15,16,17]. Recently emphasis has been placed on adopting instead heterogeneous recyclable catalyses [12,18]. So far a diversity of such catalyses were employed [19,20]. Moreover, chitosan, a chiral polysaccharide, has been used as an efficient catalyst for enantioselective syntheses that result in the creation of asymmetric products with chiral center(s) [21,22,23,24,25]. Previously, while using chitosan [9,11], we suffered from some disadvantages of this catalyst namely, that it afforded low yields of products in many of the investigated reactions and it could not easily be recycled because of its high hygroscopicity, leading it to form gels. Herein, we successfully overcome the drawbacks of chitosan and enhance its catalytic activity via heterogeneous grafting with 4-vinyl-pyridine (VP) [26] (Scheme 1), thereby producing a basic, recyclable, eco-friendly biocatalyst that could be used efficiently in Michael additions, especially when a stronger basic catalyst than chitosan, is required. Also, the use of the chitosan-g-polyvinyl pyridine, Cs-PVP in the form of beads is usually required to increase the catalytic activity of the catalyst [18,27,28]. 

**Scheme 2:**
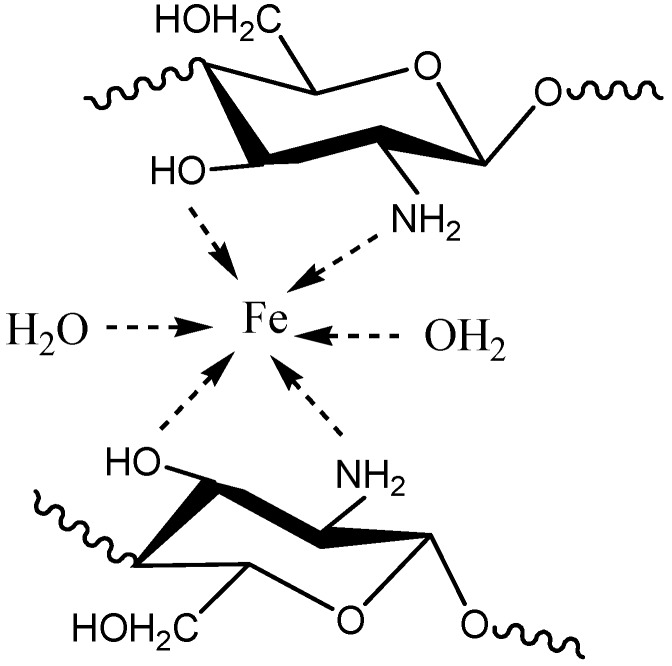
Probable structure of chelated complexes of chitosan with iron (III) ions.

In addition, chitosan has been reported to have high affinity for adsorption of metal ions [29,30,31,32]. The adsorption capacity of Fe(III) ions for chitosan/4-vinylpyridine copolymer is higher than that of the original non-grafted chitosan owing to the presence of pyridine moieties in the PVP side chains [33]. Herein, chitosan-g-poly(4-vinylpyridine)-supported iron(III) complex, [Cs-PVP/Fe(III)], could be prepared in the form of beads (Scheme 2) [34] of higher catalytic activity, and effectively used as a powerful recyclable catalyst to oxidize methylpyridazinone in the presence of hydrogen peroxide to afford the corresponding furopyridazinone derivative. The surface adsorption of Fe^3+^ ions on the surface of chitosan involves nitrogen and oxygen atoms of the repeating units of chitosan as shown below [34]:

Cs–NH_2_ + Fe^3+^ ↔ [Cs – NH_2_ --- Fe --- ]^+3^

## 2. Results and Discussion

### 2.1. Preparation of Cs-PVP Catalyst Beads

**Figure 1:**
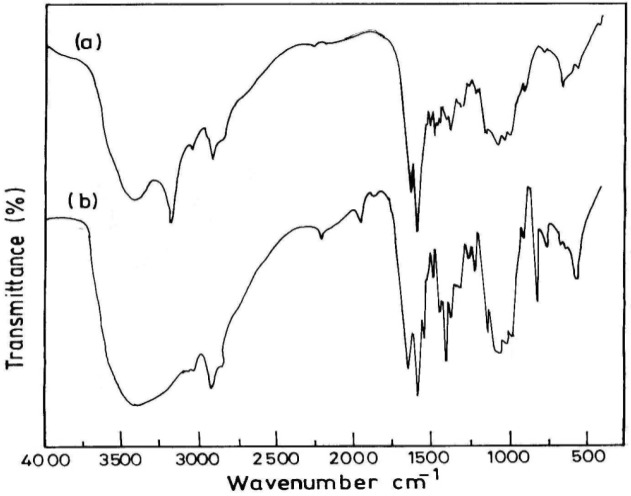
FTIR of (a) Chitosan, (b) chitosan-*g*-poly(4-vinylpyridine) copolymer 80% G.

**Figure 2:**
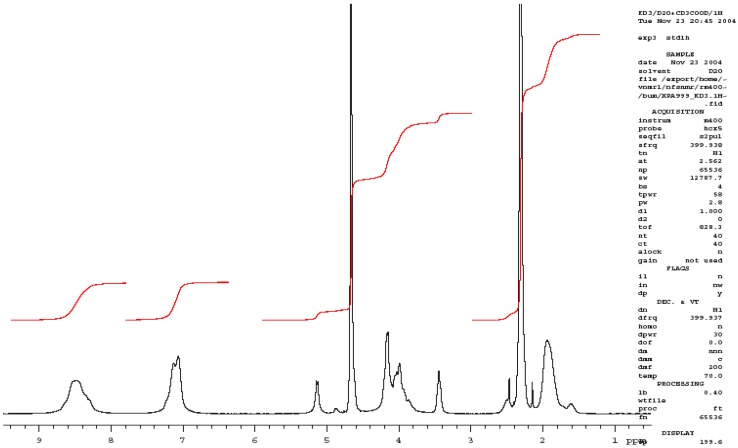
^1^H-NMR spectrum of 80% VP grafted chitosan.

The investigated basic catalyst, (Cs-PVP), was prepared through heterogeneous grafting of 4-vinylpyridine onto chitosan powder as previously reported by Khalil *et al.* [26]. The structure of grafted chitosan copolymer was confirmed by FT-IR, ^1^H-NMR, ^13^C-NMR and elemental analysis as reported in our previous work [26]. Elemental analysis showed that the nitrogen content of chitosan, N% = 7.1, was increased upon grafting with 4-vinylpyridine to N% = 8.0, which is an evidence for the presence of pyridine moieties. The structure of the grafted copolymer was confirmed by FTIR, that showed a characteristic C=N groups band at 2,197 cm^−1^ from the poly 4-vinylpyridine chains (Figure 1). Also, the presence of pyridine rings is confirmed in the ^1^H-NMR spectrum (Figure 2) by the presence of the peaks at 7.1 and 8.5 ppm in the aromatic range (Figure 2).

The prepared Cs-PVP beads exhibited more basic character (pka = 5.9), as compared to chitosan (pka = 6.4), owing to the presence of pyridine moieties along the chitosan backbone. The cross-linked chitosan graft copolymer is characterized by higher thermal stability as compared to chitosan [26] so it could then be used effectively in higher temperature reactions (TGA was reported previously [26]).

### 2.2. Applying Cs-PVP Beads as Efficient Basic Heterogeneous Catalyst in Michael Additions

**Scheme 3:**
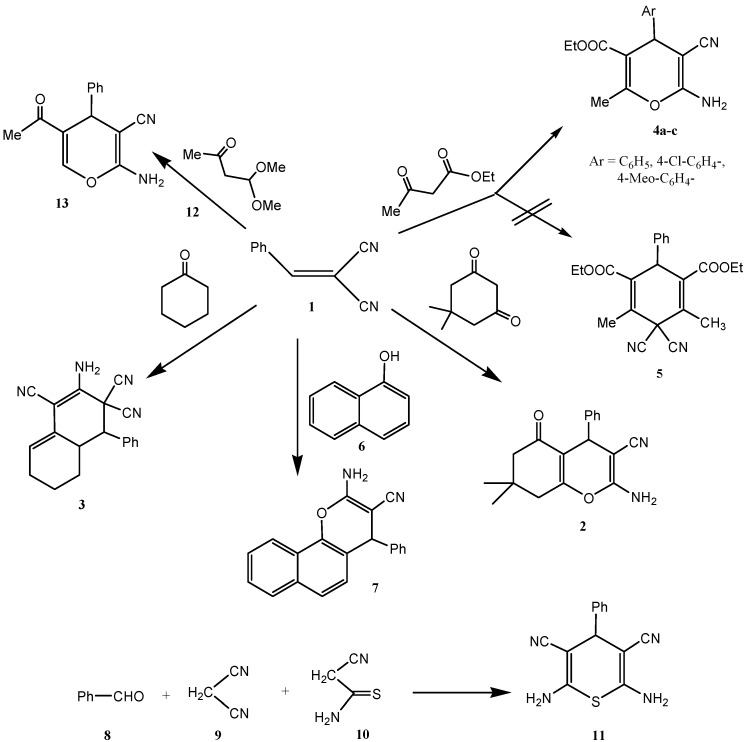
Heterogeneous basic catalyzed Michael additions using Cs-PVP beads.

**Figure 3:**
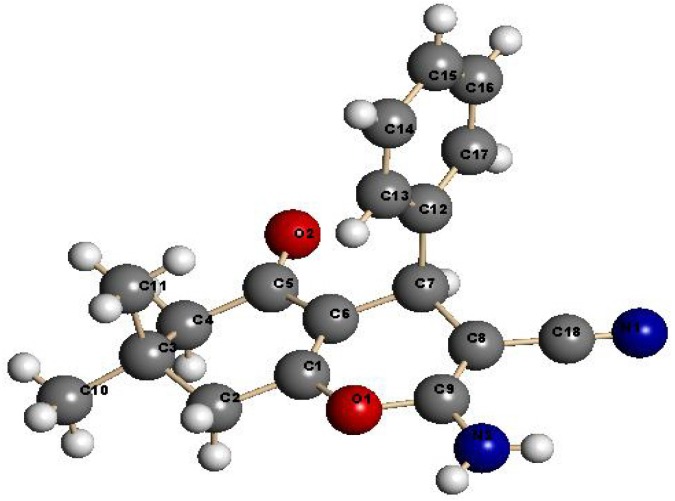
X-ray crystal structure of chromene-3-carbonitrile derivative **2**.

**Figure 4:**
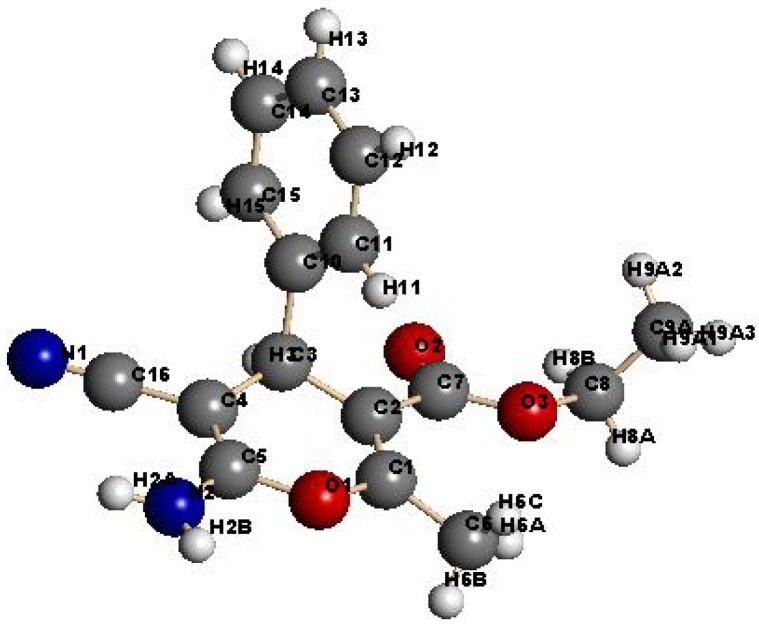
X-ray crystal structure of 2-aminopyran derivative **4**.

Benzylidene-malononitrile **1** reacted with dimedone to yield the chromene-3-carbonitrile derivative **2** in an excellent yield (>95%), more than that was obtained in presence of the original chitosan (75%), (Scheme 3). The product structure was assigned by using X-ray crystallographic analysis (Figure 3) [35]. These derivatives could be previously prepared in the presence of homogeneous basic catalysts [9,10,11,12,13,14,15,16,17]. As a result of the cross-linking nature of the invented Cs-PVP catalyst, it could be readily isolated by simple filtration and reused again for more than five times without loss of its catalytic activity, after extensive washing with hot ethanol and drying in an oven at 100 °C for 4 h. Similarly, reaction of **1** with cyclohexanone was conducted successfully in the presence of Cs-PVP beads to give a similar product **3** to that was obtained previously [9] in the presence of chitosan (55%) but in much higher yield (~90%). While the reaction of **1** with ethyl acetoacetate has been reported earlier [36] to produce the 2-aminopyran **4** together with the benzene derivative **5**, only **4** was formed in a higher yield ~90% when **1** was reacted with ethyl acetoacetate in ethanol in presence of Cs-PVP beads. Again, the produced structure was confirmed by X-ray crystal structure determination (Figure 4). The catalyst could be easily isolated from the reaction media and recycled several times after being washed with ethanol and dried in an oven. α-Naphthol **6** also reacted with **1** to yield **7** in 86% yield.

**Figure 5:**
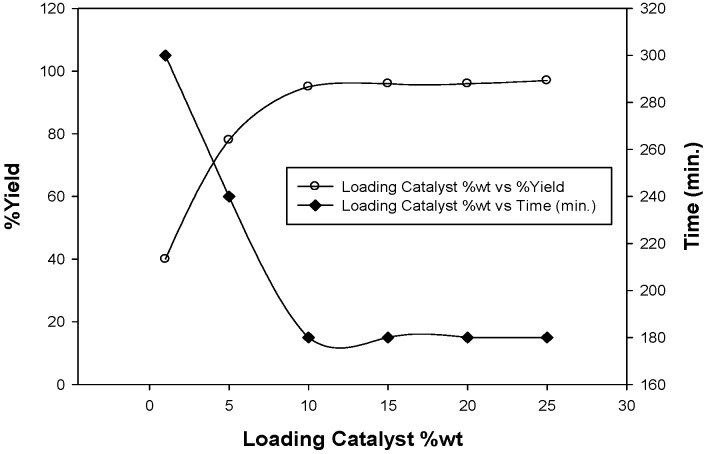
Optimization of the chitosan graft copolymer as basic catalyst.

In order to estimate the appropriate catalyst loading, a model reaction of benzylidene-malononitrile **1** (1.54 g, 10 mmol) and dimedone (1.40 g, 10 mmol) was carried out in 25 mL absolute ethanol using 1, 5, 10, 15, and 20% wt. of catalyst under the same conditions. The 10%wt catalyst loading was found to be the optimal quantity (Figure 5). Catalyst was reused four times and the results showed that the chitosan graft copolymer can be reused as such without significant loss in its catalytic activity (Table 1).

**Table 1:** Recyclability of the chitosan graft copolymer as basic catalyst.

Product	Fresh Catalyst	Recycled (1)	Recycled (2)	Recycled (3)	Recycled (4)
**2**	95	93	93	92	92

All these products were previously [9,11] obtained in presence of chitosan but in lower yields, and furthermore, with the latter, removal of the product from the catalyst could not be easily accomplished as in each case the catalyst adsorbed part of the reaction media forming a gel mass. The cross-linked graft copolymer beads did not behave similarly and its use in the form of beads has enhanced its catalytic activity to a great extent. The thiopyran **11** could be obtained in a one pot reaction of **8**, **9**, and **10**, (yields: Cs = 80%; Cs-PVP = 95%; cf. Scheme 3). This product has been obtained earlier via addition of cyanothioacetamide to **1**. While **1** failed to add to **12** in the presence of chitosan or even piperidine, it reacted smoothly with **12** in the presence of Cs-PVP beads affording the 5-acetyl-2-aminopyran derivative **13** in 80% yield.

**Scheme 4:**
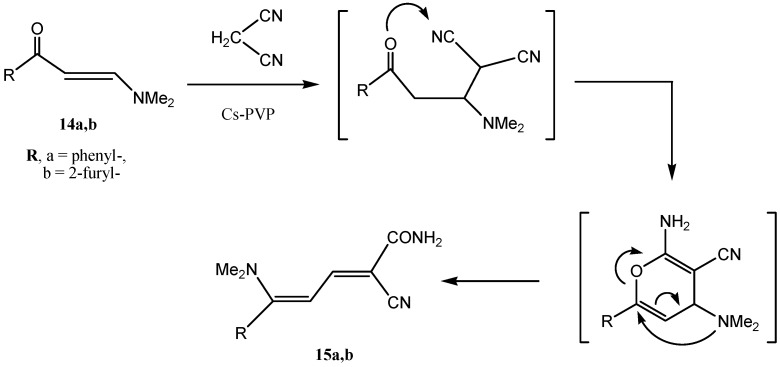
Synthesis of dieneamides by using Cs-PVP basic catalyst.

Some time ago the formation of dieneamide **15a** via reaction of enaminone **14a** with malononitrile has been reported from our laboratories [37,38]. We have recently shown that this product can be obtained in better yields by utilizing chitosan as catalyst [11]. Now we report that the dieneamide **15a** is also obtainable in higher yield in the presence of grafted chitosan Cs-PVP beads (yields: Cs = 80; Cs-PVP = 90). Moreover, the furanyl derivative **15b** could be obtained from **14b** (yields: Cs = 78; Cs-PVP = 95). The postulated mechanism of this remarkable transformation is shown below (Scheme 4).

### 2.3. Preparation of Cs-PVP/Fe(III) Catalyst Beads

**Figure 6:**
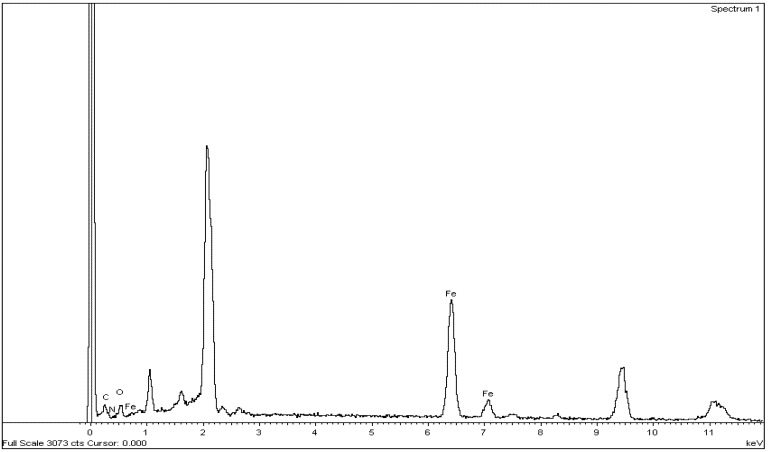
EDS of Fe(III) supported chitosan-poly(4-vinylpyridine) graft, Cs-PVP/Fe^3+^ complex.

The second goal of our study was the formation of chitosan-PVP/Fe(III) complex, Cs-PVP/Fe(III), using same methodology that published by Ngah *et al*. [33] and Burke *et al*. [34]. It is of value to mention that the previously prepared basic catalyst Cs-PVP exhibited higher metal-binding capacity, as compared to the original chitosan, owing to the presence of extra binding nitrogen-atoms with free lone pairs like in NH_2_ groups and pyridine moieties. When 50 mg of chitosan or graft chitosan were placed in a 10 nM Fe^3+^ solution at pH = 1.8, for a period of time 5 h, it was found that the adsorption capacity of the graft copolymer, Cs-PVP, was higher than that of the original chitosan, since the amount of adsorbed iron on the graft copolymer was 0.086 mmol Fe^3+^/g while it was 0.077 is for original chitosan. Elemental analysis of Fe(III)-supported chitosan graft copolymer by EDS (Table 2, Figure 6) showed that 32% of Fe(III) wascon the surface as result of the metal complexation with the N-atoms of glucosamine units and pyridine moieties and C-6 hydroxyl group binding sites over the chitosan graft surface.

**Table 2:** EDS of chitosan-*g*-poly(4-vinylpyridine)/Fe^3+^ chelate.

Element	Weight%	Atomic%
C K	17.24	38.89
N K	4.25	8.22
O K	12.25	20.74
Fe K	66.27	32.15
Totals	100.00	

**Figure 7:**
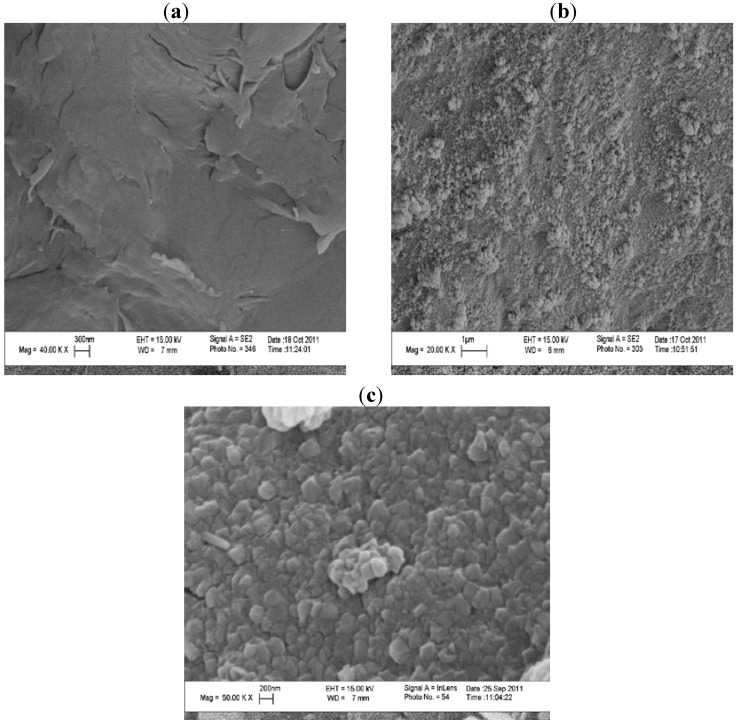
Scanning electron microscope for (**a**) Chitosan Cs, (**b**) Cs-PVP 80%G, (**c**) Cs-PVP/Fe^3+^ complex.

The produced Cs-PVP/F(III) complex could be used successfully as an efficient catalyst for oxidation of methyl pyridazinones as shown in the last part of our study. Scanning electron microscopy exhibited great differences in the surface morphology upon grafting and complexation with Fe^3+^ ions (Figure 7). From the SEM the fibrous nature of chitosan is lost upon grafting and regular geometric shapes appeared that were attributed to the coordination with Fe^3+^ ions. The suggested structure of chitosan Fe(III) chelates is supposed [39] to occur as follows (Scheme 4).

### 2.4. Utility of Cs-PVP/Fe(III) beads as an Efficient Catalyst for Oxidation of Methyl Pyridazinones

**Scheme 5:**
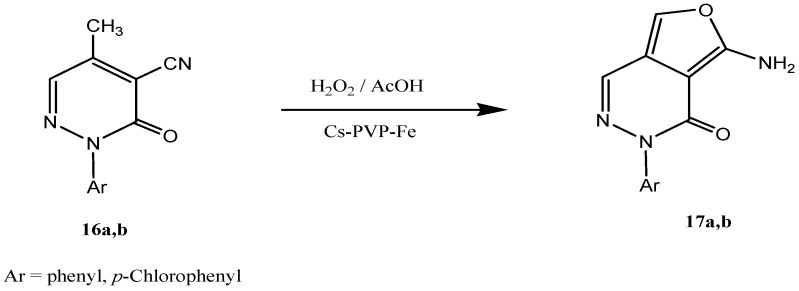
Selective oxidation of methylpyridazinones in presence of chitosan-based iron (III) catalyst, Cs-PVP/Fe(III).

One of the major challenges for organic synthesis is how to effect controlled oxidation of non-activated alkyl functions. Several approaches exist among which Fe (III) Chitosan-PVP copolymer catalysts are of utility. We would like here to report first successful controlled oxidation of the methyl function in **16** utilizing Cs-PVP/Fe (III) chelates. Thus refluxing **16a,b** with Cs-PVP/Fe (III) complex in the presence of excess hydrogen peroxide afforded **17a,b**. The potential utility of **17a,b** as precursors to phthalazines via addition of electron poor alkenes, thus replacing thienopyridazines classically utilized by Elnagdi *et al*. for this purpose will be subject of further communication (Scheme 5).

## 3. Experimental

### 3.1. General

Melting points were recorded on Gallenkamp apparatus and are reported uncorrected. The Fourier Transform infrared (FTIR) spectra were recorded in KBr pellets on a JASCO FT-IR-6300 system at a resolution of 4 cm^−1^ in the 400–4000 cm^−1^ range. ^1^H-NMR measurements were determined on a Bruker DPX spectrometer, at 400 MHz for ^1^H-NMR and 100 MHz for ^13^C-NMR, in DMSO-d_6_ as solvent using TMS as internal standard. Solid NMR spectra were recorded on a 600MHz Bruker Avance II spectrometer, operating at 150 MHz for ^13^C-NMR. Mass spectra were measured on a GC-MS DFS-Thermo spectrometer, using 70 eV EI. Elemental analyses were measured by means of an Elementar-Vario Microcube Elemental Analyzer. The scanning electron microscopy (SEM) was conducted using a JEOL JSM-630 J scanning electron microscope operated at 20 kV. (TGA data was reported previously [26]) Single crystal X-ray crystallographic analysis was performed using a Rigaku Rapid II instrument. Copies of original data can be provided upon request.

### 3.2. Samples

Chitosan, with degree of deacetylation (DDA) = 77.8%, was kindly supplied by Professor Furuhata of Tokyo Institute of Technology (T. I. Tech.). 4-Vinylpyridine, initiators (K_2_S_2_O_7_ and NaHSO_3_), and all solvents were purchased from Sigma-Aldrich Chemicals (Germany) and used without any further purification.

### 3.3. Materials

#### 3.3.1. Preparation of Chitosan Beads Cs

Chitosan beads were prepared according to the published procedure of Ngah *et al.* [30]. Chitosan solution was prepared by dissolving chitosan powder (2.00 g) in 5% (v/v) acetic acid solution (60 mL). The chitosan solution was sprayed into 0.50 M NaOH solution (500 mL) as a precipitation bath, which neutralized the acetic acid within the chitosan gel and thereby coagulated the chitosan gel as uniform spherical chitosan gel beads. A magnetic stirrer was used to stir the aqueous NaOH solution. The wet chitosan gel beads (hereafter called chitosan beads) were extensively rinsed with distilled water to remove any NaOH, filtered and finally air-dried to remove the water from the pores of the structure. The beads were then stored in distilled water and ready for use.

#### 3.3.2. Preparation of Basic Catalyst Chitosan-g-poly(vinylpyridine)(Cs-PVP) Beads

##### 3.3.2.1. Heterogeneous Grafting of 4-Vinylpyridine onto Chitosan

The grafting of 4-vinylpyridine onto chitosan was conducted using the same procedure reported in the literature [21], a typical heterogeneous method in which chitosan powder (1.00 g) was suspended in distilled water (50 mL) and mixed with 3 × 10^−3^ M of redox initiator mixture (1:0.75) K_2_S_2_O_7_ and NaHSO_3_, respectively. 4-Vinylpyridine monomer (1.05 g, 10 mmol) was added and the reaction was conducted at 55 °C for 3 h. At the end of reaction time, the graft copolymer was filtered off and washed with water and was then extensively purified with hot ethanol using the Soxhlet extraction technique to get rid of the unreacted monomer. Finally the graft copolymer (Cs-PVP), was dried. An 80% graft was obtained. The used amount in all reaction was 10% wt of the Cs-PVP catalyst.

##### 3.3.2.2. Preparation of Chitosan/PVP Copolymer Beads

Applying the same reported methodology [30], freshly prepared Cs-PVP copolymer was converted into spherical beads by dissolving graft copolymer (1.00 g) in 5% (v/v) acetic acid solution (50 mL). The graft copolymer solution was treated as usual and thereby the gel beads were coagulated. The wet grafted chitosan beads were extensively washed with distilled water to be ready for use.

#### 3.3.3. Preparation of Cs-PVP—Supported Iron(III) Complex by Adsorption of Fe^3+^ Ions on Cs-PVP Grafted Chitosan Beads Using Fe(NO_3_)_3_ [34]

The degree of adsorption was calculated based on the difference of Fe^3+^ concentration in aqueous solution before and after adsorption at a wavelength of 248 nm using an atomic absorption spectrophotometer (Perkin-Elmer 3100 Model). For Fe(III) adsorption onto Cs-PVP, a contact time of 5 h at pH 1.8 was used for stirred mixture of Cs-PVP beads (0.05 g) and Fe(III) solution (100 mL, 10 mM) in a 250 mL beaker. The amount adsorbed on the beads was calculated according to the following equation:

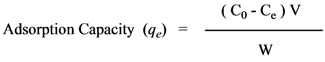

where C_0_ is the initial Fe(III) concentration (ppm), C_e_ is the final concentration of Fe(III) after the adsorption time, V is the volume of Fe(III) solution (mL) and W is the used weight of graft copolymer Cs-PVP beads (g). The yellowish-colored beads adsorbed Fe^3+^ were washed thoroughly with distilled water and stored in distilled water for further use. Under the previous conditions, the maximum adsorption capacity was obtained as 0.089 mmol Fe^3+^/g chitosan.

#### 3.3.4. Reaction of Benzylidene-Malononitrile **1** with Dimedone and Cyclohexanone

A mixture of benzylidene-malononitrile **1** (1.54 g, 10.0 mmol) with either dimedone or cyclohexanone (10.0 mmol) in absolute ethanol (50 mL) was refluxed for 3 h in the presence of a catalytic amount (10%wt.) of the catalyst (Method A using Cs beads and Method B using Cs-PVP beads). The solid product, so formed, was filtered of and recrystallized from ethanol.

*2-Amino-7,7-dimethyl-5-oxo-4-phenyl-5,6,7,8-tetrahydro-4H-chromene-3-carbonitrile* (**2**). Colorless crystals; % Yields: Method A (Cs beads = 75%), Method B (Cs-PVP beads = 95%); Mp 234 °C, lit. 233–235 °C [40]; IR (KBr): υ = 1708 (C=O), 2198 (CN), 3341, 3420 (NH_2_) cm^−1^; ^1^H-NMR (DMSO-d_6_): δ = 0.96 (s, 3H, CH_3_), 1.05 (s, 3H, CH_3_), 2.09–2.24 (s, 2H, CH_2_), 2.47 (s, 2H, CH_2_), 4.20 (s, 1H, CH, C-4 pyran), 7.01 (s, 2H, NH_2_), 7.15–7.28 (m, 5H, phenyl-H); ^13^C-NMR (DMSO-d_6_): δ = 26.9 (CH_3_), 28.5 (CH_3_), 31.9 (C(CH_3_)_2_), 35.7 (C-4, pyran), 50.1 (CH_2_), 58.5 (CH_2_), 119.8 (CN), 112.9, 126.7, 127.3, 128.5, 144.8, 158.6, 162.7 (aromatic carbons); MS, *m/z* (%), 294.1 (M^+^, 29), 217.1 (66), 55.1 (100). Anal. Calcd. for C_18_H_18_N_2_O_2_: C, 73.45; H, 6.16; N, 9.52. Found: C, 73.36; H, 6.10; N, 16.43. 

*2-Amino-4-phenyl-4a,5,6,7-tetrahydronaphthalene-1,3,3(4H)-tricarbonitrile* (**3**). Yellow crystals; % Yields: Method A (Cs beads = 55%), Method B (Cs-PVP beads = 90%); Mp 252 °C; Lit. Mp 253 °C [9]; IR (KBr): υ = 1650 (olefinic C=C), 2211 (CN), 2254 (CN) 3341, 3420 (NH_2_) cm^−1^; ^1^H-NMR (DMSO-d_6_): δ = 0.84 (m, 1H, 5-H), 1.44 (m, 2H, 5-H, 6-H), 1.66 (m, 1H, 6-H), 2.04 (m, 1H, 7-H), 2.17 (m, 1H, 7-H), 2.80 (m, 1H, 4a-H), 3.52 (d, 3*J =* 12.3 Hz, 1H, 4-H), 5.72 ("s", 1H, 8-H), 7.37 (s, 2H, NH_2_), 7.41 (m, 3H, *m*-H, *p*-H, Phenyl), 7.48 (m, 1H, *o*-H, Phenyl), 7.59 (m, 1H, *o*-H, Phenyl); ^13^C-NMR (DMSO-d_6_): δ = 21.0 (C-6), 24.9 (C-7), 27.0 (C-5), 33.8 (C-4a), 42.9 (C-3), 50.5 (C-4), 81.5 (C-1), 112.4 (3-CN), 112.6 (3-CN), 116.2 (1-CN), 120.3 (C-8), 126.9 (*o*-C, Phenyl), 128.6 (*m*-C, Phenyl), 128.8 (C-8a), 128.9 (*p*-C, Phenyl), 132.4 (*o*-C, Phenyl), 134.6 (*i*-C, Phenyl), 143.6 (C-2); MS, *m/z* (%), 300.2 (M^+^, 100), 209.1 (43), 91.1 (54).

#### 3.3.5. Reaction of Arylidene-Malononitrile with Ethyl Acetoacetate

A mixture of the arylidenemalononitrile derivative (phenyl, *p*-chlorophenyl or *p*-methoxyphenyl) (10.0 mmol) with ethyl acetoacetate (10.0 mmol) in absolute ethanol (50 mL) was refluxed for 3 h in the presence of catalytic amount (10%wt.) of the catalyst (Method A using Cs beads and Method B using Cs-PVP beads). The solid product, so formed, was filtered off and recrystallized from ethanol.

*Ethyl 6-amino-5-cyano-2-methyl-4-phenyl-4H-pyran-3-carboxylate* (**4a**). Colorless crystals; % Yields: Method A (Cs beads = 59%), Method B (Cs-PVP = 90%); Mp 194 °C; Lit. Mp 194–195 °C [41]; IR (KBr): υ = 1691 (C=O), 2190 (CN), 3329, 3385 (NH_2_) cm^−1^; ^1^H-NMR (DMSO-d_6_): δ = 1.03 (t, *J =* 7.0 Hz, 3H, ester CH_3_), 2.32 (s, 3H, pyran-CH_3_), 3.98 (AB of ABX_3_, *J =* 7.0 Hz, 2H, OCH_2_), 4.32 (s, 1H, H-4), 6.93 (br. s, 2H, NH_2_), 7.16–7.34 (m, 5H, Phenyl); ^13^C-NMR (DMSO-d_6_): δ = 13.7 (CH_3_, ester), 18.1 (CH_3_), 38.8 (C-4), 57.3 (C-3), 60.1 (CH_2_, ester), 107.3 (C-5), 119.6 (CN), 126.8 (C-4', Phenyl), 127.1 (C-2', Phenyl), 128.4 (C-3', Phenyl), 144.8 (C-1', Phenyl), 156.5 (C-6), 158.5 (C-2), 165.4 (C=O); MS, *m/z* (%), 284.1 (M^+^, 70), 207.1 (100). Anal. Calcd. for C_16_H_16_N_2_O_3_: C, 67.59; H, 5.67; N, 9.85. Found: C, 67.44; H, 5.61; N, 9.70.

*Ethyl 6-amino-4-(4-chlorophenyl)-5-cyano-2-methyl-4H-pyran-3-carboxylate* (**4b**). Pale yellow crystals; % Yields: Method A (Cs beads = 68%), Method B (Cs-PVP = 87%); Mp 162 °C; IR (KBr): υ = 1695 (C=O), 2210 (CN), 3330, 3400 (NH_2_) cm^−1^; ^1^H-NMR (DMSO-d_6_): δ = 1.10 (t, *J =* 7.1 Hz), 3H, ester CH_3_), 2.39 (s, 3H, pyran-CH_3_), 4.14 (AB of ABX_3_, *J =* 7.1 Hz), 2H, OCH_2_), 4.34 (s, 1H, 4-H), 7.01 (br. s, 2H, NH2), 7.08 (d, 2H, *o*-H, *J =* 8 Hz, aryl), 7.37 (d, 2H, *m*-H, *J =* 8 Hz, aryl); ^13^C-NMR (DMSO-d_6_): δ = 14.1 (CH_3_ of CO_2_C_2_H_5_), 19.2 (CH_3_), 37.0 (C-4), 56.1 (C-5), 61.3 (O-CH_2_), 106.5 (C-3), 118.6 (CN), 125.8, 128.7, 129.6, 146.4, 154.2, 156.8 (C-2, C-6), 167.0 (C=O); MS, *m/z* (%), 318.1 (M^+^, 37), 207.1 (100). Anal. Calcd. for C_16_H_15_ClN_2_O_3_: C, 60.29; H, 4.74; Cl, 11.12; N, 8.79. Found: C, 60.13; H, 4.66; Cl, 10.94; N, 8.57.

*Ethyl 6-amino-5-cyano-4-(4-methoxyphenyl)-2-methyl-4H-pyran-3-carboxylate* (**4c**). Colorless crystals; % Yields: Method A (Cs beads = 70%), Method B (Cs-PVP = 90%); Mp. 145 °C; Mp 144–145 °C [41]; IR (KBr): υ = 1684 (C=O), 2188 (CN), 3325, 3395 (NH_2_) cm^-1^; ^1^H-NMR (DMSO-d_6_): δ = 1.12 (t, *J =* 7.1 Hz, 3H, ester CH_3_), 2.36 (s, 3H, pyran-CH_3_), 3.79 (s, 3H, O-CH_3_), 3.99 (AB of ABX_3_, *J =* 7.1 Hz, 2H, ester CH_2_), 4.38 (s, 1H, H-4), 4.54 (br. s, 2H, NH_2_), 6.85 (d, 2H, H-3', *J =* 8 Hz, aryl), 7.16 (d, 2H, H-2', *J =* 8 Hz, aryl); ^13^C-NMR (DMSO-d_6_): δ = 18.1 (CH_3_ of ester), 37.7 (C-4), 55.0 (O-CH_3_), 60.3 (CH_2_ of ester), 62.4 (C-3), 108.1 (C-5), 114.1 (C-3'), 118.8 (CN), 128.7 (C-2'), 136.0 (C-1'), 156.4 (C-4'), 157.4 (C-6), 158.5 (C-2), 160.3 (C=O); MS, *m/z* (%), 314.1 (M^+^, 9), 184.1 (100). Anal. Calcd. for C_17_H_18_N_2_O_4_: C, 64.96; H, 5.77; N, 8.91. Found: C, 64.58; H, 5.62; N, 8.78.

#### 3.3.6. Reaction of **1** with α-Naphthol

A mixture of benzylidene-malononitrile **1** (1.54 g, 10.0 mmol) with α-naphthol (10.0 mmol) in absolute ethanol (50 mL) was refluxed for 4 h in the presence of a catalytic amount (10% wt.) of the catalyst (Method A using Cs beads and Method B using Cs-PVP beads). The solid product, so formed, was filtered off and recrystallized from ethanol.

*2-Amino-4-phenyl-4H-benzo[h]chromene-3-carbonitrile* (***7***). This compound was obtained as yellow crystals [42]; % Yields: Method A (Cs beads = 71%), Method B (Cs-PVP = 86%); Mp 210 °C; IR (KBr): υ = 1605 (Ar C=C), 1656 (olefinic C=C), 2205 (CN), 3303, 3448 (NH_2_) cm^−1^; ^1^H-NMR (DMSO-d_6_): δ = 4.90 (s, 1H, 4-H), 7.11 (d, 3*J =* 8.5 Hz, 1H, 5-H), 7.56 m, 2H/7.64, t, 1H/7.88, d, 1H/8.27, d, 1H (6-H, 7-H, 8-H, 9-H, 10-H), 7.18 (s, 2H, NH_2_), 7.24 (m, 1H, *p*-H, Phenyl), 7.25 (m, 2H, *o*-H, Phenyl), 7.32 (m, 2H, *m*-H, Phenyl); ^13^C-NMR (DMSO-d_6_): δ = 40.9 (C-4), 56.3 (C-3), 117.9, 120.7, 122.7, 123.9, 126.2, 126.6, 126.7, 126.9, 127.6, 132.7 (C-4a, C-5, C-6, C-6a, C-7, C-8, C-9, C-10, C-10a, *p*-C, Phenyl), 120.4 (CN), 127.6 (*o*-C, Phenyl), 128.7 (*m*-C, Phenyl), 142.7, 145.6 (C-10b, *i*-C, Phenyl), 160.1 (C-2); MS, *m/z* (%), 298.1 (M^+^, 75), 221.1 (100), 77 (10). Anal. Calcd. for C_20_H_14_N_2_O: C, 80.52; H, 4.73; N, 9.39. Found: C, 80.19; H, 4.64; N, 8.17.

#### 3.3.7. One Pot Reaction of benzaldehyde and malononitrile with 2-cyanothioacetamide (**11**)

A mixture of benzaldehyde (1.06 g, 10.0 mmol), malononitrile (0.66 g, 10.0 mmol) and 2-cyanothioacetamide (1.00 g, 10.0 mmol) in absolute ethanol (50 mL) was refluxed for 4 h in the presence of a catalytic amount (10% wt.) of both catalysts (Method A using Cs beads and Method B using Cs-PVP beads). The solid product was filtered off and recrystallized from ethanol.

*2,6-Diamino-4-phenyl-4H-thiopyran-3,5-dicarbonitrile* (**11**). Yellow crystals; % Yields: Method A (Cs beads = 80%), Method B (Cs-PVP = 95%); Mp 191–192 °C [36]; IR(KBr): υ = 2192 (broad band, two CN groups), 3300–3450 (two NH_2_ groups) cm^−1^; ^1^H-NMR (DMSO-d_6_): δ = 4.24 (s, 1H,4-H), 6.86–6.89 (s, 4H, two NH_2_ groups), 7.22–7.34 (m, 5H, Phenyl); ^13^C-NMR (DMSO-d_6_): δ = 43.4 (C-4), 72.0 (C-3), 118.6 (two CN), 126.7, 127.2, 128.9, 143.2 (Phenyl), 151.0 (C-2, thiopyran); MS, *m/z* (%), 254.1 (M^+^, 12), 228.1 (41), 77.1 (100). Anal. Calcd. for C_13_H_10_N_4_S: C, 61.40; H, 3.96; N, 22.03; S, 12.61. Found: C, 61.13; H, 3.88; N, 21.87; S, 12.52.

#### 3.3.8. Reaction of **1** with 4,4-Dimethoxybutan-2-one

A mixture of benzylidene-malononitrile **1** (1.54 g, 10.0 mmol) with acetylacetaldehyde, dimethyl acetal (AADMA, 10.0 mmol) in absolute ethanol (50 mL) was refluxed for 4 h in the presence of a catalytic amount (10%wt.) of the catalyst (Method A using Cs beads and Method B using Cs-PVP beads). The solid product, so formed, was filtered off and recrystallized from ethanol.

*5-Acetyl-2-amino-4-phenyl-4H-pyran-3-carbonitrile* (**13**). Yellow powder; % Yields: Method A (Cs beads = 0%), Method B (Cs-PVP = 70%); 198 °C; IR (KBr): υ = 1701 (C=O), 2205 (CN), 3320–3400 (NH_2_ groups) cm^−1^; ^1^H-NMR (DMSO-d_6_): δ = 2.32 (s, 3H, CH_3_), 4.29 (s, 1H, C-2 pyran), 6.94 (s, 2H, NH_2_), 7.14–7.33 (m, 5H, Phenyl); ^13^C-NMR (DMSO-d_6_): δ = 19.4 (CH_3_), 38.6 (C-4, pyran), 60.9 (C-3, pyran), 114.2 (C-5, pyran), 116.8 (CN), 122.1, 128.4, 129.0, 138.6, (phenyl), 152.9, 157.3 (C-2, C-6 pyran), 183.9 (C=O); MS, *m/z* (%), 240.1 (M^+^, 24), 197.1 (92), 163.1 (100). Anal. Calcd. for C_14_H_12_N_2_O_2_: C, 69.99; H, 5.03; N, 11.66. Found: C, 69.78; H, 4.92; N, 11.49.

#### 3.3.9. Reaction of Enaminones **14a,b** with Malononitrile

A mixture of (10.0 mmol) of enaminone **14a,b** with malononitrile (0.66 g, 10.0 mmol), in absolute ethanol (50 mL) was refluxed for 3 h in the presence of catalytic amounts (10%wt.) of the catalyst (Method A using Cs beads and Method B using Cs-PVP beads). The solid product, so formed, was filtered off and recrystallized from ethanol.

*2-Cyano-5-(dimethylamine)-5-phenylpenta-2,4-dienamide* (**15a**). Yellow crystals; % Yields: Method A (Cs beads = 80%), Method B (Cs-PVP = 90%); Mp: 257 °C; IR (KBr, cm^-1^): ν = 1667 (CO), 2191 (CN), 3330 and 3395 (NH_2_); ^1^H-NMR (400 MHz, DMSO_-_d_6_) δ = 2.77 (s, 3H, CH_3_), 3.16 (s, 3H, CH3), 5.63 (d, 1H, *J* = 12.6 Hz, H-4), 6.84 (s, 2H, NH_2_), 6.98 (d, 1H, *J* = 12.6 Hz, H-3), 7.26–7.29 (m, 2H, phenyl-H), 7.38–7.41 (m, 3H, phenyl-H). ^13^C-NMR (125 MHz, DMSO-d_6_), δ = 39.8 (N(CH_3_)_2_), 87.6, 96.9, 118.8 (CN), 128.6, 129.6, 134.0 (aromatic carbons), 153.3 (C-3), 164.6 (C-5), 165.11 (C=O). MS (*m/z* (%)): 241.1 (M^+^, 100), 179.1(76). Anal. Calcd. for C_14_H_15_N_3_O: C, 69.69; H, 6.27; N, 17.41. Found: C, 69.56; H, 6.27; N, 17.29.

*2-Cyano-5-(dimethylamino)-5-(furan-2-yl)penta-2,4-dienamide* (**15b**). Reddish brown crystals; % Yields: Method A (Cs beads = 78%), Method B (Cs-PVP = 95%); M.p. 245 °C; IR (KBr, cm^−1^): ν = 1669 (CO), 2189 (CN), 3340 and 3305 (NH_2_); ^1^H-NMR (400 MHz, DMSO-d_6_), δ = 2.97 (s, 6H, N(CH_3_)_2_), 5.59 (d, 1H, *J =* 12.5 Hz, H-4), 6.72 (d, 1H, *J =* 5.0 Hz, furyl H-3), 6.78 (d, 1H, *J =* 5.0 Hz, furyl H-5), 7.02 (s, 2H, NH_2_), 7.56 (d, 1H, *J =* 12.5 Hz, H-3), 7.99 (t, 1H, *J =* 5.0 Hz, furyl H-4); ^13^C-NMR (125 MHz, DMSO-d_6_): δ = 40.77, 90.40, 98.31, 111.53, 115.75, 118.38, 144.80, 145.37, 151.73, 153.40 (C-5), 164.49 (CONH_2_). MS (*m/z* (%)): 231.1 (M^+^, 21), 179.1(100). Anal. Calcd. for C_12_H_13_N_3_O_2_: C, 62.33; H, 5.67; N, 18.17. Found: C, 62.19; H, 5.57; N, 17.58.

#### 3.3.10. Oxidation of Methyl Pyridazinone with Cs-PVP-Fe(III) Complex

Hydrogen peroxide solution (10 mmol, 30%) was added carefully to a preheated solution of methylpyridazinones **16a,b** (2 mmol) in glacial acetic acid (5 mL). The reaction mixture was then refluxed in the presence of 10%wt of Cs-PVP/Fe catalyst until thin layer chromatography indicated that the reaction was completed. The solid crude product, so formed, was poured into ice/water mixture (20 mL) and stirred for 30 min. The product so formed was extracted using CH_2_Cl_2_. The organic layer was dried over anhydrous Na_2_SO_4_ and then concentrated under reduced pressure to yield analytically pure products **17a,b**. The used Cs-PVP/Fe(III) catalyst could be regenerated and used for 5 times without appreciable loss of activity.

*7-Amino-2-phenylfuro[3,4-d]pyridazin-1(2H)-one* (**17a**). Yellow powder; M.p. 198 °C; IR (KBr, cm^-1^): ν = 1660 (CO), 3330 and 3390 (NH_2_); ^1^H-NMR (400 MHz, DMSO-d_6_), δ = 6.07 (s, 2H, NH_2_), 7.46 (s, 1H, pyridazinone), 7.51 (m, 5H, phenyl), 8.14 (s, 1H, furan); ^13^C-NMR (125 MHz, DMSO-d_6_), δ = 114.1, 121.6, 126.8, 130.7, 131.2, 139.9, 140.8, 144.0, 147.2 (aromatic carbons), 168.2 (C=O). MS (*m/z* (%)): 227.1 (M^+^, 12), 77.1 (100). Anal. Calcd. for C_12_H_9_N_3_O_2_: C, 63.43; H, 3.99; N, 18.49. Found: C, 63.28; H, 3.78; N, 18.29.

*7-Amino-2-(4-chlorophenyl)furo[3,4-d]pyridazin-1(2H)-one* (**17b**). Pale yellow powder. (M.p. 216 °C); IR (KBr, cm^−1^): ν = 1668 (CO), 3360 and 3330 (NH_2_); ^1^H-NMR (400 MHz, DMSO-d_6_), δ = 6.29 (s, 2H, NH_2_), 7.25 (d, 2H, o-H, *J =* 8 Hz, aryl), 7.69 (d, 2H, m-H, *J =* 8 Hz, aryl), 8.26 (s, 1H, furan); ^13^C-NMR (125 MHz, DMSO-d_6_) δ = 113.1, 124.4, 126.9, 128.6, 129.0, 133.1, 139.1, 151.7, 155.9 (aromatic carbons), 171.9 (C=O). MS (m/z (%): 261.1 (M+, 4), 111.1 (100). HRMS; 261.0298 (C_12_H_8_O_2_N_3_Cl_1_).

## 4. Conclusions

We could clearly show that chitosan-g-PVP beads can be used as an efficient basic, recyclable, environmentally friendly biocatalyst for Michael additions while its supported iron (III) complex is a promising catalyst for controlled oxidation of alkyl functions. The novel catalysts, Cs-PVP and Cs-PVP/Fe(III) beads, could be regenerated and reactivated at least five times, without appreciable loss of activity.
